# Development of ACE2 autoantibodies after SARS-CoV-2 infection

**DOI:** 10.1371/journal.pone.0257016

**Published:** 2021-09-03

**Authors:** John M. Arthur, J. Craig Forrest, Karl W. Boehme, Joshua L. Kennedy, Shana Owens, Christian Herzog, Juan Liu, Terry O. Harville

**Affiliations:** 1 Department of Medicine, University of Arkansas for Medical Sciences, Little Rock, AR, United States of America; 2 Central Arkansas Veterans Healthcare System, Little Rock, AR, United States of America; 3 Department of Microbiology and Immunology, University of Arkansas for Medical Sciences, Little Rock, AR, United States of America; 4 Center for Microbial Pathogenesis and Host Inflammatory Responses, University of Arkansas for Medical Sciences, Little Rock, AR, United States of America; 5 Winthrop P. Rockefeller Cancer Institute, University of Arkansas for Medical Sciences, Little Rock, AR, United States of America; 6 Department of Pediatrics, University of Arkansas for Medical Sciences, Little Rock, AR, United States of America; 7 Department of Pathology and Laboratory Services, University of Arkansas for Medical Sciences, Little Rock, AR, United States of America; Max Delbruck Centrum fur Molekulare Medizin Berlin Buch, GERMANY

## Abstract

**Background:**

Activation of the immune system is implicated in the Post-Acute Sequelae after SARS-CoV-2 infection (PASC) but the mechanisms remain unknown. Angiotensin-converting enzyme 2 (ACE2) cleaves angiotensin II (Ang II) resulting in decreased activation of the AT1 receptor and decreased immune system activation. We hypothesized that autoantibodies against ACE2 may develop after SARS-CoV-2 infection, as anti-idiotypic antibodies to anti-spike protein antibodies.

**Methods and findings:**

We tested plasma or serum for ACE2 antibodies in 67 patients with known SARS-CoV-2 infection and 13 with no history of infection. None of the 13 patients without history of SARS-CoV-2 infection and 1 of the 20 outpatients that had a positive PCR test for SARS-CoV-2 had levels of ACE2 antibodies above the cutoff threshold. In contrast, 26/32 (81%) in the convalescent group and 14/15 (93%) of patients acutely hospitalized had detectable ACE2 antibodies. Plasma from patients with antibodies against ACE2 had less soluble ACE2 activity in plasma but similar amounts of ACE2 protein compared to patients without ACE2 antibodies. We measured the capacity of the samples to inhibit ACE2 enzyme activity. Addition of plasma from patients with ACE2 antibodies led to decreased activity of an exogenous preparation of ACE2 compared to patients that did not have antibodies.

**Conclusions:**

Many patients with a history of SARS-CoV-2 infection have antibodies specific for ACE2. Patients with ACE2 antibodies have lower activity of soluble ACE2 in plasma. Plasma from these patients also inhibits exogenous ACE2 activity. These findings are consistent with the hypothesis that ACE2 antibodies develop after SARS-CoV-2 infection and decrease ACE2 activity. This could lead to an increase in the abundance of Ang II, which causes a proinflammatory state that triggers symptoms of PASC.

## Introduction

SARS-CoV-2 causes a spectrum of symptoms collectively known as COVID-19 and can range from asymptomatic infection to severe disease. Both symptomatic and asymptomatic COVID-19 patients can have long lasting symptoms after the infection has cleared [1]. The long-lasting effects have been termed “Long Covid” but more recently, the syndrome is referred to as Post-Acute Sequelae of SARS-CoV-2 infection (PASC). The cause of these symptoms is unknown. Since acute symptoms are not required to develop PASC, the cause is not likely due to direct tissue injury related to infection. Many of the manifestations of acute COVID-19 are caused by overactivation of the immune system rather than direct effects of the virus on host tissue [2]. One proposed mechanism for activation of the immune system acutely is by induction of the renin angiotensin system. The enzyme ACE2 is the viral receptor for the SARS-CoV-2 virus and is expressed as both a membrane bound and a soluble form. The biological function of ACE2 is to convert the octapeptide angiotensin II (Ang II) to angiotensin (1–7). Ang II binds to the AT1 receptor to produce immune activation and other effects [3, 4]. Ang (1–7) binds to the Mas receptor to decrease inflammation and produce other effects [5]. Thus, the presence of higher levels of ACE2 protein decreases the effects mediated by activation of the AT1 receptor including immune activation (i.e., increased ACE2 activity results in decreased inflammation). Binding of SARS-CoV-2 to ACE2 results in decreased activity of the enzyme [6]. The net result is increased inflammation during SARS-CoV-2 infection. The immune system also is implicated in sequelae after SARS-CoV-2 infection. For instance, antinuclear [7], antiphospholipid [8] and antiinterferon [9] antibodies have been found after infection. While the renin angiotensin system (RAS) could also be involved in immune activation in the chronic setting, a mechanism for immune activation by RAS has not been described. One possibility is that persistent shedding of ACE2 results in lower total amounts of the enzyme. Persistent shedding occurs for at least 35 days after acute infection [10] and is associated with decreased activity of membrane bound ACE2 [11]. Antibodies against ACE2 were previously identified in patients with connective tissue diseases, and IgG purified from plasma of these patients can inhibit ACE2 activity [12]. We hypothesized that an autoantibody against ACE2 develops after SARS-CoV-2 infection. This antibody could decrease the activity of both soluble and membrane bound ACE2 leading to activation of receptors for Ang II and activation of the immune system. We used samples from patients with a history of SARS-CoV-2 infection and controls to show that an autoantibody against ACE2 is present in some patients after infection, that patients that have ACE2 antibodies have lower amounts of soluble ACE2 activity, and that plasma from these patients can inhibit ACE2 activity.

## Methods

### Cohort

Human samples and data were analyzed under a non-human subjects determination by the UAMS Institutional Review Board. Samples were obtained from residual samples at the clinical lab at UAMS hospital or from donated plasma. They were deidentified prior to access by the researchers. They were not part of an existing bank. Remnant plasma or serum samples were collected from inpatients at UAMS hospital with a positive PCR test for SARS-CoV-2 (15 patients) and from outpatient clinics (33 patients). Remnant samples were collected from blood drawn for clinical purposes and that were planned for disposal after five days of refrigeration. Of the outpatients, 20 had a positive SARS-CoV-2 PCR virus test and 13 had a negative test. We also obtained 32 plasma samples from plasma donors to be used for convalescent plasma treatment. These samples were obtained from patients that had a known positive virus test by PCR and had been symptom free for at least two weeks prior to donation of plasma. There were no other inclusion or exclusion criteria. We refer to these groups as: Inpatient+, Outpatient+, Outpatient–and Convalescent+.

### ELISA assays for SARS-CoV-2 and ACE2 antibodies

Fifty microliters of a solution of recombinant SARS-CoV-2 receptor binding domain protein (2 μg/mL, plasmid from BEI) or recombinant ACE2 protein (2 μg/mL, SinoBiologicals) in carbonate buffer (0.0125 M Na_2_CO_3_, 0.0875 M NaHCO_3_, pH 9.4) was added to each well of a high binding ImmunoGrade 96-well plate (MidSci) and coated overnight. To determine the presence and concentration of antibodies in plasma or serum, samples were diluted 1:50 in 1% dry milk PBS-T (1X PBS, 0.1% Tween-20) and added to duplicate wells for 2 hours, followed by peroxidase-conjugated goat anti-human IgG + IgM antibody (JacksonImmuno) diluted at 1:5000 in 1% dry milk PBS-T. Seventy-five microliters of a solution containing tetramethyl benzidine (SeraCare—SureBlue TMB Solution) was added and stopped after 5 minutes with 75 μL of 1% HCl solution (SeraCare-TMB stop solution). The optical density at 450 nm was determined. The value of a blank well control was subtracted to obtain the final value and reported as OD (450 nm). All measurements were made in duplicate and the mean value of the two wells was used for the analysis. Cutoff values for a positive test were defined as mean of negative controls plus 3 standard deviations. The cutoff values are 0.60 for the RBD antibody and 0.1106 for ACE2 antibody.

### ELISA assay for soluble ACE2 protein in plasma

We used an ELISA from Raybiotech (Norcross, GA) to measure the concentration of soluble ACE2 protein in plasma following the manufacturer’s instruction. Briefly, samples were diluted 8-fold and loaded in duplicates on 96 well plates precoated with an antibody against human ACE2. Plates were incubated for 2.5 hours, washed, and incubated with a biotinylated detection antibody for 1 hour. Plates were washed and incubated with HRP conjugated streptavidin solution for 45 min. Plates were washed and incubated with TMB (tetramethylbenzidine) One Step Substrate Reagent for 30 min. Reaction was stopped with 0.2M sulfuric acid and absorbance measured at 450 nm. A serial dilution of recombinant ACE-II was loaded in duplicates and served as standard curve. ACE2 concentrations (ng/ml) were calculated based on a standard curve using 4 parametric nonlinear regression.

### Activity assay for ACE2 enzyme activity in plasma

We used a fluorometric activity assay from Sigma (St. Louis, MO) to measure the activity of soluble ACE2 enzyme in plasma following the manufacturer’s instruction: Plasma samples were diluted 1:5 in ACE2 Lysis Buffer. 5μl were incubated with 45 μl of assay buffer for 15 min. 50 μl of ACE2 substrate mix containing a peptide-MCA (4-methylcoumarin-7-acetate) conjugate was added and the change in fluorescence recorded (Ex 320 nm/Em 420 nm) over 30 min on a SpectramaxM5 (Molecular Devices: San Jose, CA). Lysis Buffer was used as background control, recombinant ACE2 used as positive control. To distinguish ACE2 activity from other proteolytically active enzymes, activity of samples was also measured in the presence of a specific ACE2 inhibitor. For the negative control ACE2 inhibitor was added to positive controls. ACE2 activity of the samples was calculated based on a standard curve created by recording fluorescence of serial dilutions of MCA at Ex320nm/Em420nm.

### ACE2 inhibitor assay

We used the ACE2 inhibitor screening assay kit from AMSBIO (Cambridge, Mass) to measure the ability of plasma or serum from patients in our cohorts to inhibit ACE2 activity. The manufacturer’s instructions were followed. The assay uses a fluorescent substrate that is an Ang II analog. Cleavage of the substrate by ACE2 removes the 2,4-dinitrophenyl moiety that quenches the fluorescence of the 7-methoxycoumarin moiety, resulting in increased fluorescence [13]. We added 5 μL of plasma or serum to the assay with a final volume of 50 μL. Percent inhibition was determined by comparing the control condition to the well with plasma or serum added. All measurements were made in duplicate and the mean value of the two wells was used for the analysis.

### Statistical analysis

All data used for this manuscript are in the supporting data table called S1 Table. Comparisons were made between the patients that had an ACE2 antibody level above the cutoff threshold and the patients that did not have an ACE2 antibody level above the threshold. Statistical comparison of these groups was done using a by Mann-Whitney test. Comparison of differences between the mean values of groups for antibody concentration and percent change of ACE2 activity caused by addition of plasma was done using a one-way ANOVA followed by Tukey-Kramer test. Error bars represent standard error of the mean.

## Results

**Fig 1:**
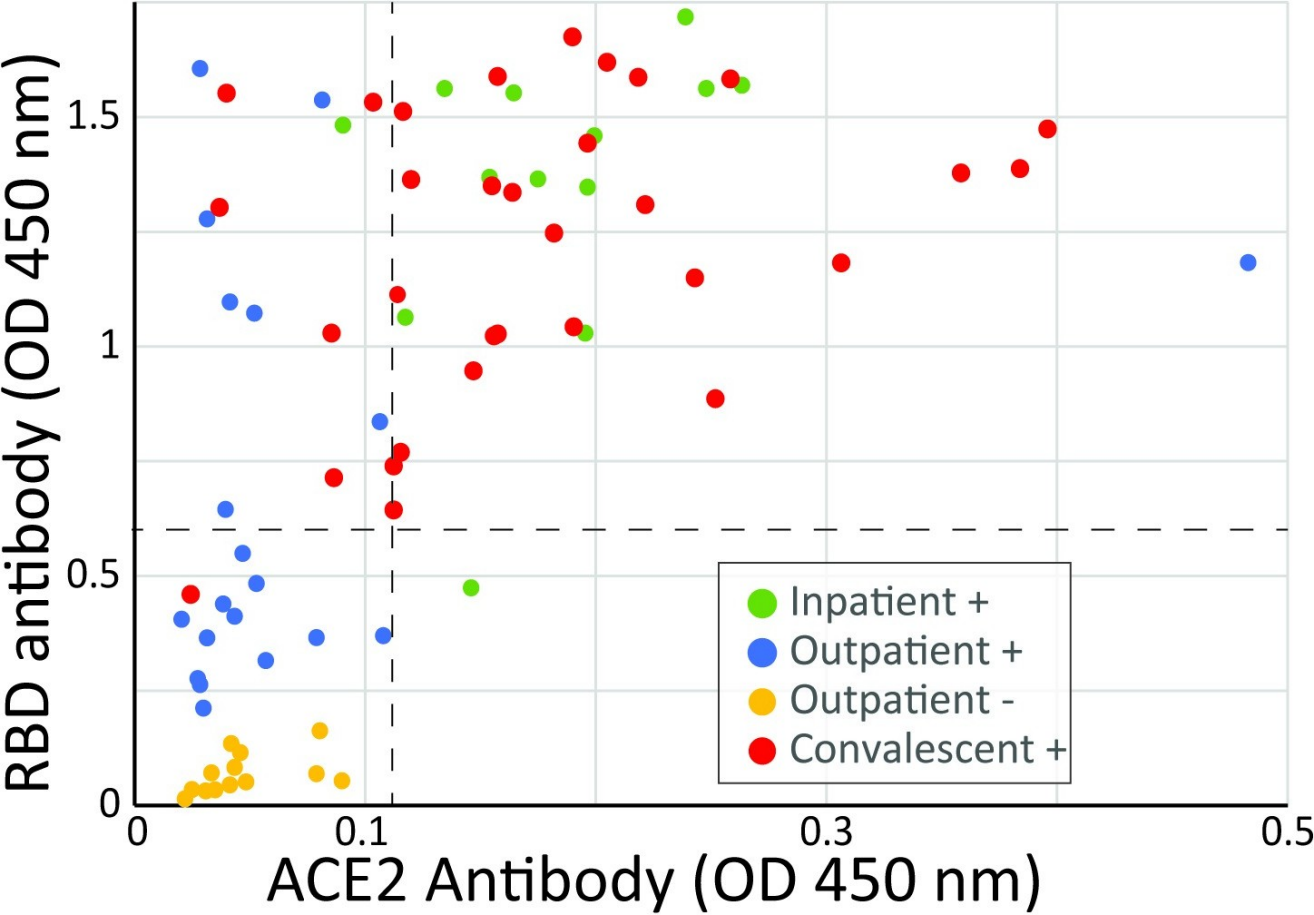
Abundance of antibodies that recognize the SARS-CoV-2 RBD and ACE2 protein in plasma or serum. The dotted line represents the cut-off values for a positive antibody test that was defined as the value of the mean of negative controls plus 3 standard deviations.

Antibody against the SARS-CoV-2 RBD was present in 93% of the Inpatient+ group and 97% of the Convalescent+ patients but in only 40% of the Outpatient+ group and none of the patients in the Outpatient- group (Fig 1). Antibody against ACE2 was present in 93% of Inpatient+ and 81% of Convalescent+ patients but was below the cutoff value in all but one of the Outpatient+ and all of the Outpatient- group. The mean Optical densities for both the Inpatient+ and Convalescent+ groups were statistically different from the Outpatient+ and the Outpatient- groups (P<0.01). Overall, of the 27 patients that had RBD antibody abundance below the cutoff level, only one (4%) had ACE2 antibody. Of the 53 patients that had RBD antibody above the cutoff value, 40 (75%) had ACE2 antibody. These data indicate that development of SARS-CoV-2-specific antibodies correlates with the development of antibodies against ACE2. For further analysis, we divided the patients into a group that had ACE2 antibodies above the cutoff threshold (+, 41 patients) and those that did not have antibodies above the threshold (-, 39 patients). The number of patients that did and did not have ACE2 antibodies from each of the locations where samples were collected is shown in Table 1.

**Table 1:** 

	Inpatient +	Outpatient +	Outpatient -	Convalescent +	Total
ACE2 Antibody +	14	1	0	26	**41**
ACE2 Antibody -	1	19	13	6	**39**
**Total**	**15**	**20**	**13**	**32**	

**Fig 2:**
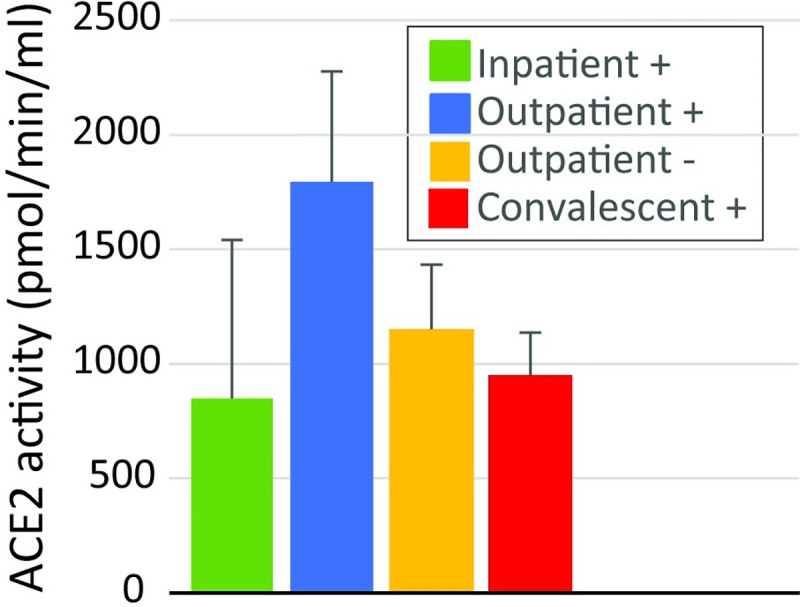
ACE2 enzyme activity in the four collection groups. There was no statistically significant difference between any of the groups.

**Fig 3:**
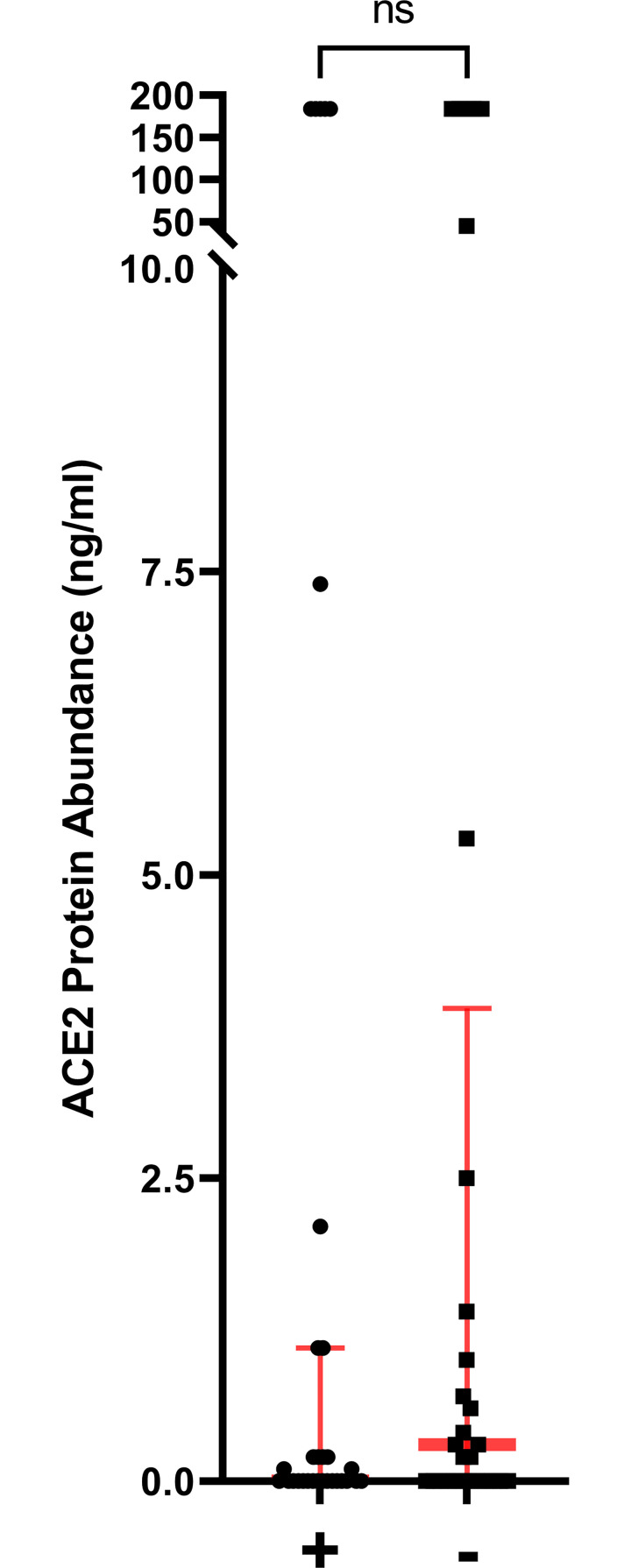
Abundance of soluble ACE2 protein in plasma of patients that had ACE2 antibodies in plasma compared to those that did not. There was no statistically significant difference in ACE2 protein between groups. The group labeled + had ACE2 antibodies and the group labeled–did not have ACE2 antibodies. (ns = nonsignificant).

**Fig 4:**
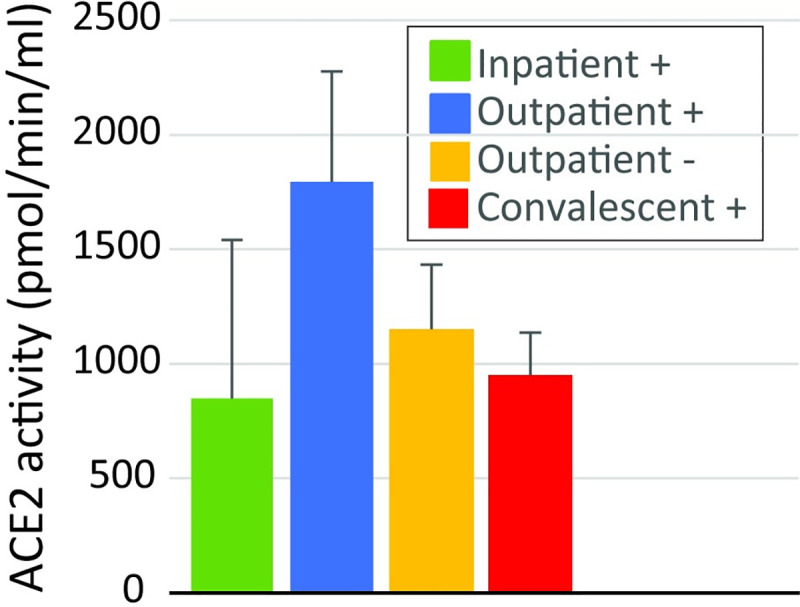
Activity of soluble ACE2 protein in plasma of patients that had ACE2 antibodies in plasma compared to those that did not. Activity of the enzyme in plasma was decreased in patients that had an ACE2 antibody present. Bars show mean values and error bars show standard error of the mean (** p<0.01). The group labeled + had ACE2 antibodies and the group labeled–did not have ACE2 antibodies.

We compared the amount of soluble ACE2 protein in plasma and the activity of soluble ACE2 in plasma. There were no differences in ACE2 protein concentration in plasma. There was also no statistically significant difference in soluble ACE2 activity in plasma when we compared the collection groups (Fig 2). When we compared the 41 patients that had an ACE2 antibody to the 39 that did not, there was no statistical difference in the abundance of ACE2 protein in the plasma between the group of patients that did and did not have an ACE2 antibody (Fig 3). The median abundance of the group with an ACE2 antibody was 0 ng/ml (IQR 0.0–1.1) and the median value of the group that did not have an ACE2 antibody was 0.3 ng/ml (IQR 0–3.9). In contrast, the activity of soluble ACE2 in the plasma of patients that had an ACE2 antibody was lower than the activity for patients that did not have an ACE2 antibody (Fig 4, p<0.01). The median activity of soluble ACE2 in patients with an ACE2 antibody was 263 pmol/min/ml (IQR 0–1039) compared to 1056 (IQR 457–2230) for those that did not have an antibody.

**Fig 5:**
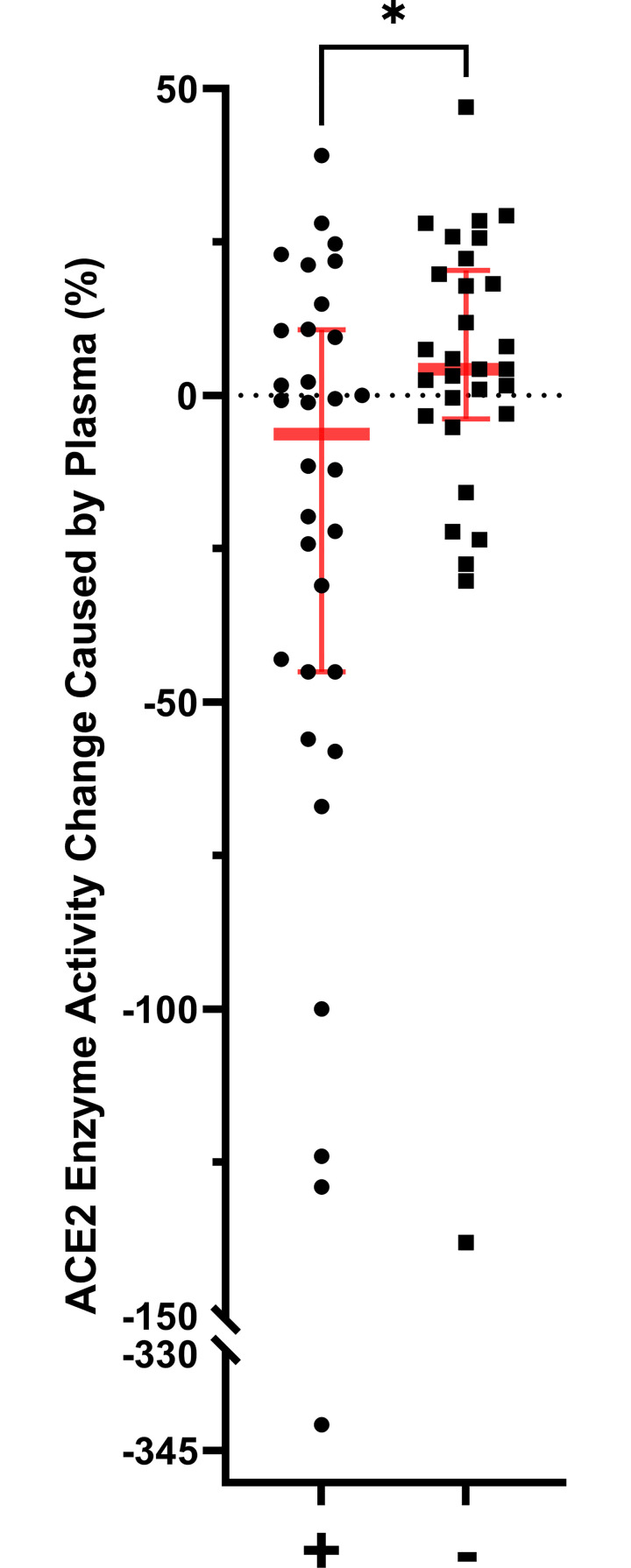
Change in activity of exogenous ACE2 after addition of plasma from patients that had ACE2 antibodies in plasma (+) compared to those that did not (-). Plasma from patients with an ACE2 antibody present decreased ACE2 activity. Bars show mean values and error bars show standard error of the mean (*** p<0.05).

**Fig 6:**
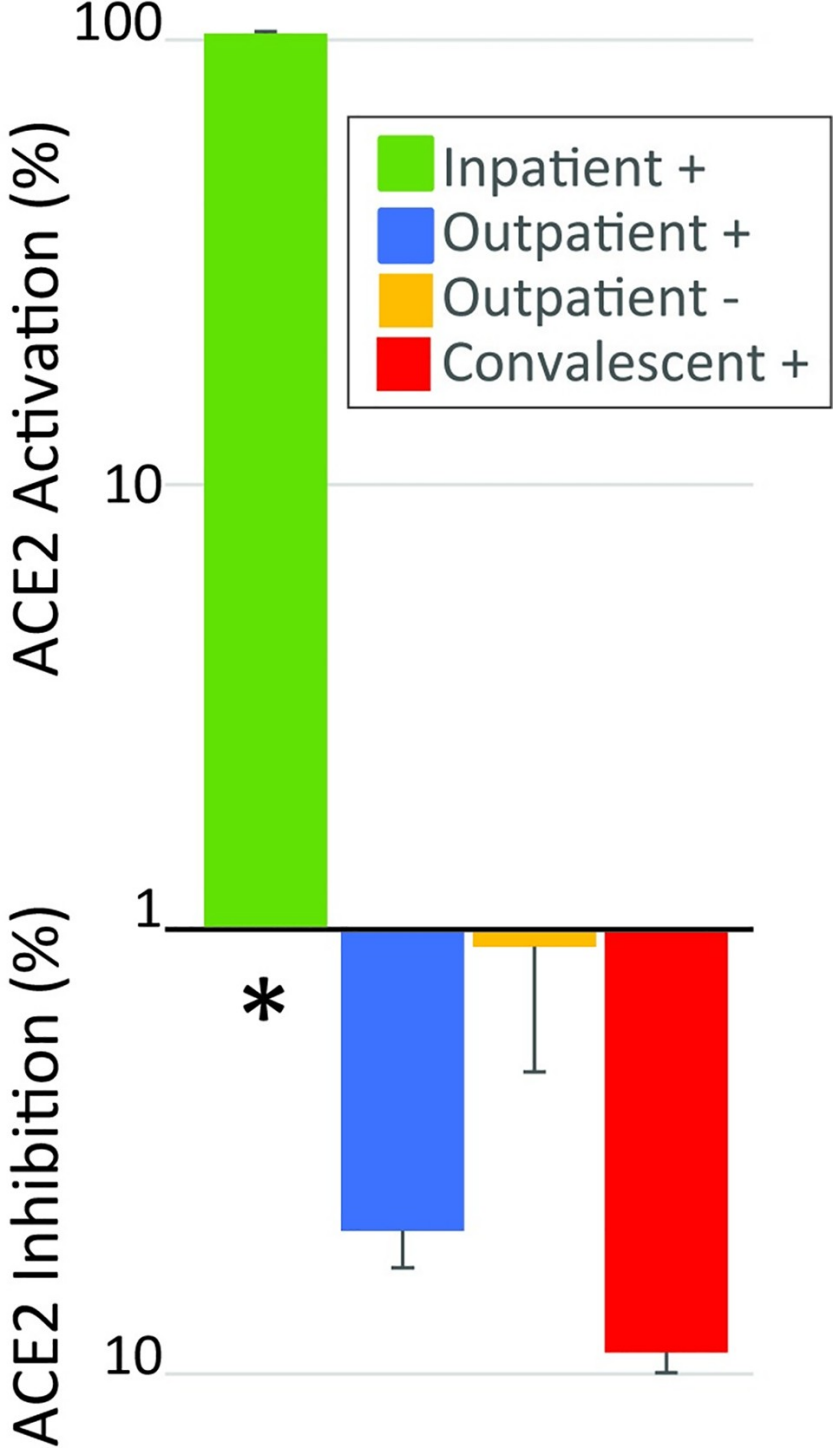
Mean change in ACE2 activity between groups of patients after addition of plasma or serum to the activity assay. Addition of plasma caused activation in the inpatient + group. * p<0.01 vs all other groups.

**Fig 7:**
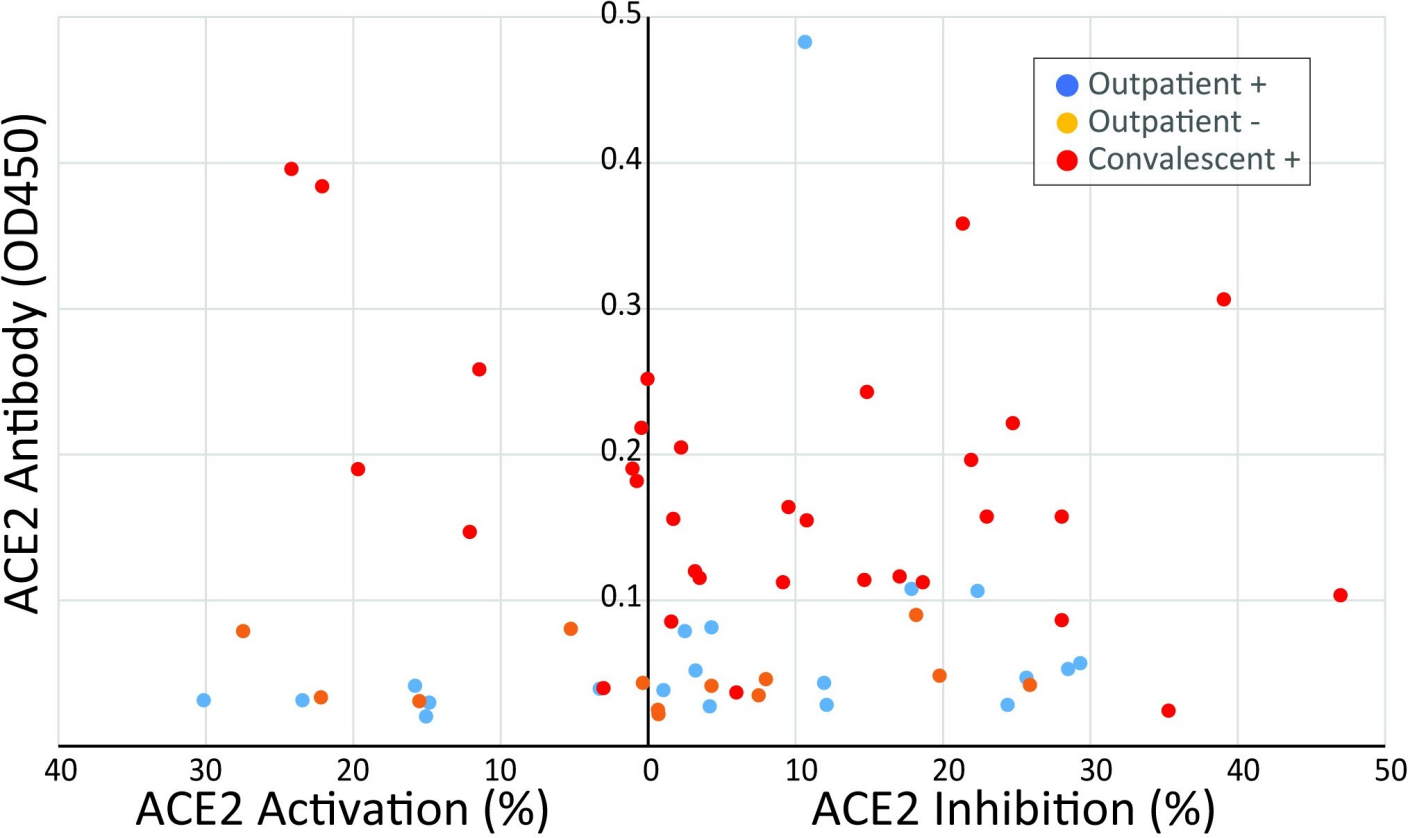
Inhibition of ACE2 activity by plasma. The abundance of the ACE2 antibody is plotted against the change in ACE2 activity after addition of plasma.

To determine if addition of plasma that contained an ACE2 antibody was associated with inhibition of ACE2 enzyme activity, we added plasma to an assay in which an exogenous source of ACE2 and its substrate was present (Fig 5). We compared the activity of the assay after plasma from the 41 patients that had an antibody above the threshold level was added to the activity of the assay when plasma from the 39 patients that did not have ACE2 antibody concentration above the threshold was added. Addition of plasma that contained an ACE2 antibody inhibited ACE2 enzyme activity compared to addition of plasma without an antibody (-6.3, IQR-45.0 to 10.8% vs 4.3, IQR -3.8 to 20.4%, p<0.05). In addition to comparing the groups with and without antibodies, we compared the ability of plasma from each of the four collection groups to alter the activity of the exogenous ACE2 assay. Addition of plasma or serum from the Inpatient+ group resulted in activation of ACE2 activity (102 ± 23%), which was statistically different (P<0.01) from all other groups (Figs 6 and 7).

## Discussion

We found that ACE2-specific antibodies are present in patients after SARS-CoV-2 infection. These antibodies may develop early in the disease process since they were detected in 93% of the patients hospitalized for COVID-19. Interestingly, only one of the twenty outpatients with a known SARS-CoV-2 infection had ACE2 antibodies. It is possible that these patients had not yet had time to develop ACE2 antibodies, However, because we used residual samples that had been deidentified, we do not know the timing of infection relative to sample collection. This is the first demonstration of anti-ACE2 antibodies in patients with SARS-CoV-2 infection. It is likely that the early antibodies are IgM, and the later ones are IgG, although our assay did not differentiate between these subtypes. Since anti-ACE2 antibodies were detected almost exclusively in patients that have formed antibodies against the RBD of SARS-CoV-2, it is likely that these are anti-idiotypic antibodies. The difference in the percent of subjects with ACE2 antibodies could be due to timing of sample collection relative to the infection, but it could also be due to severity of illness. Wang et al. demonstrated that COVID-19 patients exhibit increases in autoantibodies compared to healthy controls and that patients with more severe disease develop higher levels of autoantibodies [14].

Anti-idiotypic antibodies are antibodies that are specific to the antigen-binding region of a host antibody that recognizes a foreign protein [15]. In this case, antibody 1 is the host antibody that recognizes the viral RBD protein. Antibody 2 is a host anti-idiotypic antibody that recognizes the binding domain of antibody 1. Some of these antibodies also recognize the binding partner of the original viral protein. In this case, the binding partner is the host ACE2 protein. This subset of anti-idiotypic antibodies are called internal image [16] or homobodies [17]. Thus, after developing an antibody that recognizes the RBD of SARS-CoV-2, the host can develop an antibody that recognizes and potentially inhibits its own ACE2 enzyme. This is one mechanism by which viruses trigger autoantibodies that cause autoimmune diseases [18]. We speculate that the autoantibodies seen in these patients may be anti-idiotypic antibodies. In SARS-CoV-2 infection, they may be relatively common since an antibody against ACE2 was present in 93% of the Inpatient+ and 81% of Convalescent+ patients in our cohort.

Three unresolved issues regarding the response to SARS-CoV-2 can potentially be explained using Jerne’s Network Theory of the Immune System [16]. First, as we have discussed, the formation of anti-idiotypic antibodies to the SARS-CoV-2 spike protein could result in anti-ACE2 antibodies, as part of the normal homeostasis of immune system function. Second, there are anecdotal data suggesting that patients experiencing PASC, who become vaccinated with a SARS-CoV-2 vaccine may have improvement. This is also aligned with Jerne’s Network Theory, as the vaccine may induce the immune system to balance the idiotypic and anti-idiotypic antibodies for homeostatic control. Third, from early in the COVID-19 disease process there are reports that anti-SARS-CoV-2 antibodies last for only a few months. This again is consistent with Jerne’s Theory, in that immunologic control mechanisms should typically limit production of an autoimmune antibody, which could result in disease. Thus, the idiotype/ anti-idiotype interactions, with the anti-idiotype having autoimmune potential, could result in down-regulation of the idiotypic antibody (homeostatic balancing). Since the half-life of IgG is 21–28 days, significant loss of anti-SARS-CoV-2 antibody responses over 6–9 months is plausible.

The majority of Ang II conversion to Ang (1–7) in tissues such as the kidney is due to membrane bound ACE2 [19]. Tissue-bound ACE2 can be cleaved and circulates in plasma in a soluble form, however, most of the ANG II conversion in plasma is due to the enzyme prolyloligopeptidase and to a lesser extent prolylcarboxypeptidase [19]. We measured the plasma concentration of ACE2 protein and the activity of soluble plasma ACE2 to determine if there was a difference in these measurements between patients with and without ACE2 antibodies. Recently, there have been reports of increases in soluble ACE2 activity in patients with acute or recovered COVID-19. A case report of a single patient with severe COVID-19 showed that the patient had increased ACE2 activity peaking at about 40-fold higher than normal levels on about day 10 and gradually declining thereafter [20]. A second study showed that soluble ACE2 activity was elevated in recovered SARS-CoV-2 patients a median of 35 days after infection compared to healthy controls [10]. Moreover, ACE2 activity was higher in the group with more severe COVID-19 compared to those with milder disease. In contrast, our study did not show a change in soluble ACE2 activity in any of the collection groups relative to the negative control (Fig 2). When we compared soluble ACE2 activity between patients that did and did not have ACE2 antibodies, we saw a decrease (Fig 4). The reason for these differences is unclear although the timings of collection may have been different and the study from Patel [10] compared SARS-CoV-2 patients to healthy controls whereas our control group was patients without SARS-CoV-2 infection who likely had other diseases processes. However, none of these studies including ours examined tissue bound ACE2, which is likely the more physiologically relevant source of ANG II conversion to Ang (1–7).

The studies of soluble ACE2 and ACE2 activity in plasma reflect the potential effect of an inhibitor on ACE2 which has been shed from membrane-bound sources, but the results can be confounded by differences in the magnitude of shedding of the enzyme. To attempt to determine if the ACE2 antibody in plasma correlates with an ability to inhibit the activity of ACE2 enzyme, we used an assay with exogenous sources of ACE2 and substrate. Addition of plasma from patients with ACE2 antibodies inhibited exogenous ACE2 activity relative to addition of plasma from patients without antibodies (Fig 5). This suggests that the ACE2 antibodies in the plasma may inhibit the ACE2 activity. This inhibition would likely affect ACE2 enzyme that is tissue-bound as well as the activity of soluble ACE2. This provides a potential mechanism for alteration of the balance of angiotensin peptides leading to increased Ang II and activation of the immune system. Thus, we have two lines of evidence that support the hypothesis that anti-idiotypic antibodies lead to PASC symptoms. The first is the presence of ACE2 antibodies after SARS-CoV-2 infection that are not found in patients that have not been infected. The second is the finding that addition of plasma that contains these antibodies can decrease ACE2 activity. Although these findings are suggestive, they do not prove causation.

When we analyzed the result by the patient group instead of by presence of an ACE2 antibody, we found that ACE2 activity is significantly increased when plasma or serum from hospitalized patients with acute COVID-19 (Inpatient+) is added to the activity assay. This activation of ACE2 activity by addition of plasma is consistent with the effect of an ACE2 activator in the plasma of these acutely ill patients that may be able to overcome the inhibition by ACE2 antibody. Regulation of the expression of ACE2 is a major modifier of the activity of the enzyme [21] but exogenous small molecule activators of ACE2 have also been identified [22, 23] suggesting that there could be endogenous molecules that activate ACE2 as well. To date, no endogenous ACE2 activators have been described. The finding of activation of ACE2 by plasma from the inpatient + groups is consistent with the study from Patel and colleagues who showed increased ACE2 activity in plasma after SARS-CoV-2 infection [10]. Since many patients appear to develop ACE2 antibodies after infection with SARS-CoV-2 but a smaller fraction develop long term symptoms, there may be differences in the ability of ACE2 autoantibodies to inhibit the enzymatic activity. There is large amount of variability in the correlation between ACE2 antibody levels and activation or inhibition of ACE2 enzyme activity (Fig 6), even among the Convalescent+ patients (shown in red dots). This variability could be due to two factors. First, there could be a competing substance in plasma or serum that causes activation of ACE2 activity. Second, even though the antibodies can recognize ACE2, they may not all inhibit its enzymatic activity. If this is correct, patients represented by the dots farthest to the right on Fig 6 are most likely to develop symptoms of PASC.

Limitations of this study include the (i) use of deidentified samples so a correlation with PASC symptoms could not be determined, (ii) a relatively small sample size, and (iii) the inability of our assay to distinguish between IgG and IgM ACE2 antibodies. Moreover, the study does not prove a causal relationship between anti-ACE2 antibodies and symptoms of PASC as we do not have any data regarding PASC symptoms in this cohort. Nevertheless, the finding that ACE2 antibodies are present after infection with SARS-CoV-2 and that plasma from patients with antibodies can inhibit ACE2 activity provides a potential mechanism for PASC.

These studies show for the first time that ACE2 antibodies are present after SARS-CoV-2 infection. This finding is consistent with a hypothesis that ACE2 antibodies may be involved in a process that leads to immune activation. While we do not have data about the association of ACE2 antibodies and PASC in this cohort, we hypothesize that antibodies could initiate a cascade of effects that lead to the symptoms of PASC. If these antibodies are responsible for symptoms of PASC, several treatments are possible. Angiotensin receptor blockers are safe and widely used. These drugs would mitigate the effects of increased Ang II caused by inhibition of ACE2. An association between protection from sequelae of SARS-CoV-2 infection and treatment with angiotensin receptor blockers or ACE inhibitors has not yet been examined but should be a high priority for ongoing research into PASC. Treatment with these RAS blockers may not be possible however in patients with low blood pressure. More targeted therapy of the mechanism of ACE2 inhibition is possible. Recombinant soluble ACE2 protein is proposed as a treatment during acute phases of infection but may also be useful for PASC. Small molecule activators of ACE2 are available and have been proposed for treatment of hypertension and may be useful for the treatment of PASC [22, 23]. Thus if the relationship between ACE2 antibodies and PASC is confirmed, several treatments will be available.

## Supporting information

S1 Table The table shows the data for RBD antibody concentration, ACE2 antibody concentration, ACE2 protein concentration in plasma, ACE2 activity level and inhibition of ACE2 activity with addition of plasma. The collection group of each sample is listed.
(XLSX)
