# Correction: Development of ACE2 autoantibodies after SARS-CoV-2 infection

**DOI:** 10.1371/journal.pone.0314426

**Published:** 2024-11-20

**Authors:** John M. Arthur, J. Craig Forrest, Karl W. Boehme, Joshua L. Kennedy, Shana Owens, Christian Herzog, Juan Liu, Terry O. Harville

In the Discussion section, there is an error in the last sentence of the sixth paragraph. The correct sentence is: First, there could be a competing substance in plasma or serum that causes activation of ACE2 activity. Second, even though the antibodies can recognize ACE2, they may not all inhibit its enzymatic activity. If this is correct, patients represented by the dots farthest to the right on Fig 7 are most likely to develop symptoms of PASC.
